# Application of the Food Quality Protection Act children’s health safety factor in the U.S. EPA pesticide risk assessments

**DOI:** 10.1186/s12940-020-0571-6

**Published:** 2020-02-10

**Authors:** Olga V. Naidenko

**Affiliations:** Environmental Working Group, 1436 U St NW, Suite 100, Washington DC, 20009 USA

**Keywords:** Children’s health safety factor, Food Quality Protection Act, Pesticides, Risk assessment

## Abstract

**Background:**

The Food Quality Protection Act of 1996, or FQPA, required the Environmental Protection Agency to set allowable levels for pesticides in a way that would “ensure that there is a reasonable certainty that no harm will result to infants and children from aggregate exposure to the pesticide chemical residue.” The act stipulated that an additional tenfold margin of safety for pesticide risk assessments shall be applied to account for pre- and postnatal toxicity and for any data gaps regarding pesticide exposure and toxicity, unless there are reliable data to demonstrate that a different margin would be safe for infants and children.

**Discussion:**

To examine the implementation of the FQPA-mandated additional margin of safety, this analysis reviews 59 pesticide risk assessments published by the EPA between 2011 and 2019. The list includes 12 pesticides used in the largest amount in the U.S.; a group of 35 pesticides detected on fruits and vegetables; and 12 organophosphate pesticides. For the non-organophosphate pesticides reviewed here, the EPA applied an additional children’s health safety factor in 13% of acute dietary exposure scenarios and 12% of chronic dietary exposure scenarios. For incidental oral, dermal and inhalation exposures, additional FQPA factors were applied for 15, 31, and 41%, respectively, of the non-organophosphate pesticides, primarily due to data uncertainties. For the organophosphate pesticides as a group, a tenfold children’s health safety factor was proposed in 2015. Notably, in 2017 that decision was reversed for chlorpyrifos.

**Conclusions:**

For the majority of pesticides reviewed in this study, the EPA did not apply an additional FQPA safety factor, missing an opportunity to fully use the FQPA authority for protecting children’s health.

## Background

A recent study published in *Environmental Health* concluded that the U.S. has lagged behind other agricultural nations in banning harmful pesticides and suggested that pesticide bans can catalyze the transition to safer alternatives [1]. A similarly important question is whether, in the current practice of pesticide registrations in the U.S., all measures are taken to protect the public, especially infants and children, from the potentially harmful effects of pesticides. The Food Quality Protection Act (FQPA) of 1996 (Public Law 104–170) is considered a landmark pesticide legislation because of its requirement to ensure a “reasonable certainty that no harm will result to infants and children from aggregate exposure to the pesticide chemical residue.” The FQPA passage led to restrictions on certain neurotoxic insecticides, including limitations on residential and agricultural spraying of organophosphate insecticides; lower levels of organophosphate residues on produce; and a 70% decline in the use of organophosphates between 2000 and 2012 in the United States [2].

The FQPA stipulated that “an additional tenfold margin of safety for the pesticide chemical residue and other sources of exposure shall be applied for infants and children to take into account potential pre- and postnatal toxicity and completeness of the data with respect to exposure and toxicity to infants and children.” The act also stated, “notwithstanding such requirement for an additional margin of safety, the Administrator may use a different margin of safety for the pesticide chemical residue only if, on the basis of reliable data, such margin will be safe for infants and children.” Initially, the tenfold children’s health safety factor was viewed as a key component in pesticide risk assessment. In a 2002 policy guidance on the determination of the appropriate FQPA safety factors, the EPA referred to “a presumption in favor of applying an additional 10X safety factor”, and a factor greater than 10X was also considered as an option [3].

**Fig. 1:**
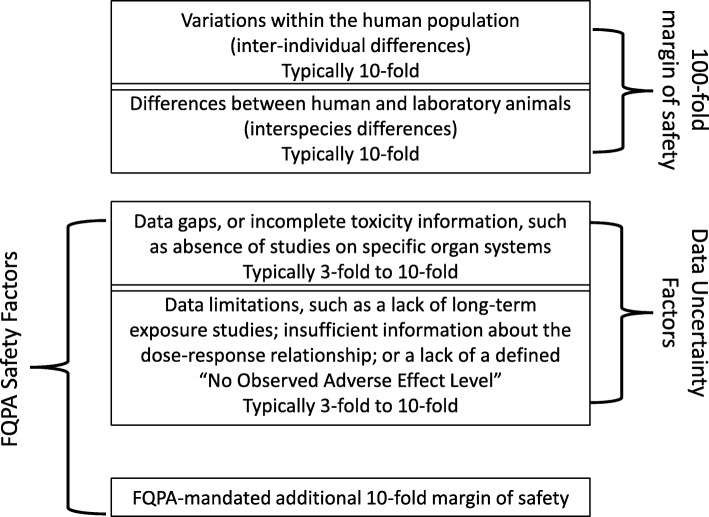
Relationship between the FQPA safety factors and other safety and uncertainty factors used in pesticide risk assessment. Graphic based on the reports by the EPA [3, 4] and the National Research Council [5, 6]

Figure 1 demonstrates the relationship between various uncertainty factors and the FQPA children’s health safety factor within pesticide risk assessments [3–6]. As described in numerous research publications and two reports published by the National Research Council in 2006 [5] and in 2009 [6], such assessments typically use a tenfold uncertainty factor to account for inter-species variations, or extrapolation from animal studies to humans; and another tenfold uncertainty factor to account for differences within the human population (Fig. 1, upper portion of the chart). This practice reflects the concept of a 100-fold margin of safety, which dates back to 1954 [7]. Research over the past two decades pointed out that the tenfold factors for interspecies and intraspecies differences may not fully represent the range of sensitivities within the human population which can be greater than tenfold [6]. Yet, these two default factors remain in practice and are used in the EPA pesticide risk assessments. Together with the 100-fold margin of safety, risk assessments can also include data uncertainty factors that address data gaps and limitations in the existing studies [3, 4]. The FQPA introduced an additional 10-fold margin of safety, and the FQPA-mandated safety assessment applies both to the children’s health safety factor and to other sources of uncertainty (Fig. 1, lower portion of the chart) [3, 4].

The application of an additional children’s health factor in pesticide risk assessments was infrequent from the start of FQPA implementation, as the U.S. Government Accounting Office (now called Government Accountability Office) reported in 2000 [8]. In 2001, the Consumers Union reported that of 82 pesticide safety determinations for organophosphate insecticides made in the first 5 years following the FQPA passage, a tenfold children’s health safety factor was applied in only 13 decisions (16%) [9]. The EPA Office of Inspector General stated in 2006 that the EPA “has primarily measured its success and the impact of FQPA by adherence to its reregistration schedule rather than by reductions in risk to children’s health” [10]. In the same year, the National Research Council reported that, of 59 pesticides reviewed, EPA “found it unnecessary to apply an FQPA factor—that is, it uses a factor of 1—for all but 11 chemicals” [5]. The report added that for the five pesticides where the EPA applied a tenfold FQPA factor, “severe developmental toxicity end points, such as multiple malformations and fetal death, were observed in laboratory animals” [5]. The latest analysis of the FQPA implementation was published in 2013, when the Government Accountability Office examined the EPA’s safety factor decisions made between 1996 and 2012 and found that in 22% of those decisions, the EPA applied the default tenfold factor [11]. Thus, a new review of the EPA’s implementation of the FQPA safety factor is both timely and needed.

### Selection of pesticides for the analysis

Pesticides selected for this analysis comprise three groups. The first group, consisting of 12 pesticides used in the largest volume in U.S. agriculture (Table 1), was identified from U.S. Geological Survey data for 2016, the latest year available [12]. The second group consists of 35 pesticides detected on fruits and vegetables in testing conducted by the U.S. Department of Agriculture between 2016 and 2018 (Table 2) [25]. The third group consists of 12 organophosphate insecticides that have been reviewed by the EPA since 2015 (Table 3).

**Table 1:** Pesticides used in the largest volume in the U.S. agriculture

Pesticide name and type, ordered by volume of use	Estimated use in 2016, millions of pounds [12]	EPA document year	The FQPA factor for acute dietary exposures	The FQPA factor for chronic dietary exposures	Reference
Glyphosate (herbicide)	288–290	2017	N/A	1X	[13]
Atrazine (herbicide)	75–76	2018	1X	1X^b^	[14]
Metolachlor and S-metolachlor, combined (herbicide)	66–72	2019	N/A	1X	[15]
Dichloropropene (fumigant)	48–59	2013	N/A	1X	[16]
Acetochlor (herbicide)	45–48	2018	1X	1X	[17]
2,4-Dichlorophenoxyacetic acid (herbicide 2,4-D)	44–47	2017	1X	1X	[18]
Pendimethalin (herbicide)	11–18	2017	1X	1X	[19]
Paraquat (herbicide)	9–14	2019	1X	1X	[20]
Chlorothalonil (fungicide)	10–11	2012	N/A	1X	[21]
Glufosinate (herbicide)	9–11	2012	1X^a^	10X	[22]
Dicamba (herbicide)	8–10	2016	1X	1X	[23]
Ethephon (plant growth regulator)	9	2015	1X	1X	[24]

*N/A* not available, indicating that an FQPA factor was not assigned for this exposure scenario

^a^The EPA did not assign an FQPA factor for the acute dietary exposure scenario for glufosinate for the general population, including infants and children; for acute dietary exposure assessment for females 13–49 years of age, the EPA assigned a 1X FQPA factor

^b^Chronic dietary exposure to atrazine metabolite hydroxyatrazine

**Table 2:** Pesticides detected on fruits and vegetables

Pesticide name and type, in alphabetical order	Foods where over 30% of the samples tested between 2016 and 2018 carried detectable pesticide residues [25]	EPA document year	The FQPA factor for acute dietary exposure	FQPA factor for chronic dietary exposures	Reference
Acetamiprid (neonicotinoid insecticide)	Apples, applesauce, cherries, frozen cherries, strawberries, frozen strawberries	2017	1X	1X	[26]
Ametoctradin (fungicide)	Spinach	2017	not assigned^a^	not assigned^a^	[27]
Azoxystrobin (fungicide)	Green onions, kale, raisins	2018	3X	1X	[28]
Bifenthrin (pyrethroid insecticide)	Kale (29.7% of samples tested in 2017), raisins, frozen strawberries	2019	1X	N/A	[29]
Boscalid (fungicide)	Frozen cherries, grapes, raisins, strawberries, frozen strawberries	2018	N/A	1X	[30]
Captan (fungicide, detected by the presence of its metabolite tetrahydrophthalimide)	Applesauce, strawberries, frozen strawberries	2018	1X^b^	1X	[31]
Chlorantraniliprole (insecticide)	Cilantro, spinach	2019	N/A	1X	[32]
Chlorpropham (herbicide and plant growth regulator / potato sprout suppressant)	Potatoes	2017	N/A	10X	[33]
Clothianidin (neonicotinoid insecticide)	Spinach	2017	1X	1X	[34]
Cypermethrin (pyrethroid insecticide)	Frozen spinach	2019	1X	N/A	[29]
Cyprodinil (fungicide)	Grapes, strawberries	2016	1X	1X	[35]
DCPA (herbicide, also called dacthal and chlorthal-dimethyl)	Cilantro, kale	2011	N/A	1X	[36]
Dimethomorph (fungicide)	Spinach	2015	10X	1X	[37]
Diphenylamine (plant growth regulator)	Apples, applesauce	2018	N/A	1X	[38]
Fenbuconazole (fungicide)	Frozen cherries	2019	1X^b^	1X	[39]
Fenhexamid (fungicide)	Cherries, grapes	2018	N/A	1X	[40]
Fenpropathrin (pyrethroid insecticide)	Canned olives	2019	1X	N/A	[29]
Flonicamid (insecticide)	Spinach, frozen strawberries	2019	N/A	1X	[41]
Fludioxonil (fungicide)	Apples, cherries, pears, strawberries	2017	N/A	1X	[42]
Fluopicolide (fungicide)	Kale, spinach	2017	N/A	1X	[43]
Fluxapyroxad (fungicide)	Strawberries	2016	1X	1X	[44]
Imazalil (fungicide)	Grapefruit, oranges	2018	1X^b^	1X	[45]
Imidacloprid (neonicotinoid insecticide)	Cilantro, lettuce, canned tomatoes (29.9% of samples tested in 2017), potatoes, raisins, spinach	2017	1X	1X	[46]
Iprodione (fungicide)	Cherries	2012	10X^c^	10X	[47]
Mandipropamid (fungicide)	Spinach, frozen spinach	2018	N/A	1X	[48]
Metalaxyl/Mefenoxam (fungicide)	Cucumbers (29.8% of samples tested in 2016)	2016	1X	N/A	[49]
Permethrin, cis and trans (pyrethroid insecticide)	Spinach	2019	1X	N/A	[29]
Propamocarb (fungicide)	Cucumbers	2019	1X	1X	[50]
Pyraclostrobin (fungicide)	Frozen cherries, grapes, raisins, strawberries	2018	1X	1X	[51]
Pyrimethanil (fungicide)	Apples, pears	2015	1X	1X	[52]
Spinetoram (insecticide)	Spinach	2018	N/A	1X	[53]
Tebuconazole (fungicide)	Cherries, frozen cherries, grapes, raisins	2018	3X	3X	[54]
Thiabendazole (fungicide)	Apples, applesauce, grapefruit, mangos, oranges, pears	2019	1X	1X	[55]
Thiophanate-methyl (fungicide, detected by the presence of its metabolite carbendazim)	Applesauce, frozen strawberries	2014	1X^b^	3X	[56]
Trifloxystrobin (fungicide)	Frozen cherries, raisins	2018	1X^b^	1X	[57]

*N/A* not available, indicating that an FQPA factor was not assigned for this exposure scenario

^a^According to the EPA, “based on a review of the available ametoctradin toxicological studies, no toxicological points of departure where selected for ametoctradin and thus, an additional Food Quality Protection Act (FQPA) safety factor to protect children is not needed. As a result, no dietary, residential, occupational, or aggregate exposure assessments are required at this time” [27]

^b^The EPA did not assign an FQPA factor for the acute dietary exposure scenario for the general population, including infants and children; for acute dietary exposure assessment for females 13–49 years of age, the EPA assigned a 1X FQPA factor

^c^For iprodione, the EPA did not assign an FQPA factor for the acute dietary exposure scenario for the general population including infants and children; for acute dietary exposure assessment for females 13–49 years of age, the EPA assigned a 10X FQPA factor

**Table 3:** Organophosphate pesticides

Pesticide, ordered by volume of use	Estimated use in 2016, millions of pounds [12]	EPA document year, if 2015 or later	The FQPA factor	Reference
Chlorpyrifos	4.6–7.9	2017	1X	[58]
Acephate	3.4–4.4	2018	10X	[59]
Tribufos	2.7–2.9	2015	10X	[60]
Dicrotophos	1.04–1.06	2015	10X	[61]
Malathion	0.94–1.35	2016	10X	[62]
Phorate	0.81–0.95	Not identified	–	–
Bensulide	0.7–0.71	2016	10X	[63]
Phosmet	0.66–0.84	2016	10X	[64]
Dimethoate	0.62–1.42	2015	10X	[65]
Terbufos	0.49–2.26	2015	10X	[66]
Ethoprop (ethoprophos)	0.23–0.88	2015	10X	[67]
Naled	0.23–0.29	Not identified	–	–
Diazinon	0.069–0.073	2016	10X	[68]
Chlorethoxyfos	0.014–0.105	2016	10X	[69]

To confirm which pesticides are used in the largest volume in U.S. agriculture, the 20 most-used pesticides were first identified from USGS data [12]. From that group, sulfur, sulfuric acid, and petroleum oil were excluded as chemical substances that have extensive industrial and other uses outside of the agricultural market. The fumigants metam and metam potassium were excluded, because the EPA considers them non-food chemicals that are not subject to FQPA review [70]. Chloropicrin was excluded because there are no established tolerances for chloropicrin on food, and therefore, according to the EPA, the FQPA does not apply to this pesticide [71]. The herbicides metolachlor and S-metolachlor were combined for the purposes of this analysis. The fumigant dichloropropene has several isomers, with 1,3-dichloropropene being the most common, sold under the trade name Telone. The USGS pesticide estimates are reported for “dichloropropene” without specification of the isomer. For subsequent review, this study focused on 1,3-dichloropropene. The USGS database uses two models for estimating pesticide use, EPest-low and EPest-high [12], and both estimates are included in Table 1, rounded to a whole number. For ethephon, both high and low use estimates correspond to around 9 million.

Next, this study reviewed pesticides detected on fruits and vegetables in testing conducted by the USDA between 2016 and 2018 [25]. The USDA tests different types of produce in different years, and a three-year time frame gives a more complete overview of pesticide occurrence on produce. Pesticides detected on 30% or more of the samples were selected for the analysis in this study. Three pesticides detected on more than 29.5% of the tested samples were also included: bifenthrin, flonicamid and metalaxyl. The USDA testing is conducted on fruits and vegetables that are washed and, if needed for a specific type of produce, peeled prior to laboratory analyses [25]. Thus, these test results represent pesticide residues that would be directly ingested with the diet. The detection of pesticides on produce does not mean that the sample(s) violate the EPA’s legal limits for pesticides. Rather, the USDA test data are a reflection of current use patterns for pesticides in fruit and vegetable production in the U.S.

Third group included organophosphates with active EPA registrations for which (1) the latest USGS data indicated that over 0.01 million pounds were applied annually [12]; and (2) an EPA review published in 2015 or later could be identified on the regulations.gov website.

The FQPA regulations require the EPA to review each registered pesticide every 15 years [72]. The latest EPA documents with FQPA determinations were identified from the EPA website and the materials posted on the U.S. government website Regulations.gov. Not all documents reviewed here represent the finalized human health risk assessments. Reviewed documents also include draft assessments posted for public comments; scoping documents in support of pesticide registration reviews; Federal Register publications designating pesticide tolerances (allowable levels of pesticide on specific foods); and, in the case of chlorpyrifos, an EPA document with a denial of a public petition. While some of the reviewed documents may not constitute the final EPA decision on the FQPA factor application for a particular pesticide, those documents reflect the EPA’s view on the FQPA factor determination, even in a draft form, and are therefore included in the present analysis.

Special cases are noted for the following pesticides. For the herbicide atrazine, the FQPA determination is for chronic dietary exposure to the atrazine metabolite hydroxyatrazine, with the FQPA factor of 1X [14]. For the four pyrethroid insecticides analyzed in this study (bifenthrin, cypermethrin, fenpropathrin, permethrin), the FQPA factor determination is based on a policy document released in July 2019, which stated that “the total FQPA safety factor for pyrethroids can be reduced to 1X for all populations” [29]. For the fungicide metalaxyl, the EPA did not assess a chronic dietary exposure scenario, and this pesticide is not included in the FQPA summary statistics for chronic dietary exposures presented in this study. Metalaxyl is included in the analysis of FQPA determinations for other scenarios (acute dietary exposure; incidental oral exposure; and inhalation exposure, all with a 1X FQPA factor) [49]. For the fungicide ametoctradin, the EPA did not conduct dietary, residential, occupational, or aggregate exposure assessments [27], and thus this pesticide was not included in further analysis in this study.

### The FQPA factor determinations for dietary exposures to non-organophosphates

Of the 47 non-organophosphate pesticides reviewed in this study, the EPA evaluated acute dietary exposure scenarios for 31 chemicals and chronic dietary exposure scenarios for 41 chemicals (Tables 1 and 2). Among those, an additional FQPA factor was applied for acute dietary exposures for 4 pesticides (13% of reviewed scenarios): dimethomorph (10X); iprodione (10X); azoxystrobin (3X); and tebuconazole (3X). Additional FQPA factor was applied for chronic dietary exposures for 5 pesticides (12% of reviewed scenarios). For the remaining dietary exposure scenarios reviewed here, no additional children’s health safety factor was applied, an approach that the EPA describes as an FQPA factor of 1X. Of the five pesticides with an added FQPA factor for chronic dietary exposures, three pesticides have a 10X FQPA factor (chlorpropham, glufosinate and iprodione) and two have a 3X FQPA factor (tebuconazole and thiophanate-methyl). The Additional file 1 accompanying this article quotes the EPA explanations for the assignment of additional FQPA factors. These rationales focus on data uncertainties, such as the use of a LOAEL instead of a NOAEL for setting the reference dose (azoxystrobin, dimethomorph, glufosinate, iprodione and tebuconazole); uncertainty regarding potential thyroid toxicity (chlorpropham); and a missing study (thiophanate-methyl).

In light of the recent report by Donley on the differences in pesticide oversight in the U.S. and other countries [1], it is notable that the three pesticides with a 10X FQPA safety factor for chronic dietary exposures have use restrictions in the European Union and are classified as “not approved” in the EU pesticide database as of January 2020 [73]. In animal studies, chlorpropham was associated with an increase in Leydig cell tumors in the testes of male rats and the potential for endocrine disruption [74, 75]. Glufosinate shows “critical indications of neurotoxicity” and changes in brain enzyme function and brain morphometrics; these effects were observed in the offspring at the lowest dose of glufosinate tested, and no “No Observed Adverse Effects Level” dose was identified [22]. Neurotoxic effects of glufosinate were also reported in the peer-reviewed scientific literature [76, 77]. Finally, iprodione acts as an antiandrogenic compound, causing effects such as delayed onset of male puberty, altered anogenital distance, abnormal sperm, reductions in serum testosterone levels and persistence of areolas; it also increases the incidence of tumors in different species and organs, including interstitial Leydig cell tumors in rats and ovary luteomas and liver cell tumors in mice [47, 78, 79]. The EPA has classified iprodione as “Likely to be Carcinogenic to Humans” [47].

Overall, review of the three pesticides with a 10X FQPA factor for chronic dietary exposures is reminiscent of the conclusion of the National Research Council which stated that the EPA applied the 10X FQPA factor in a small number of cases where severe developmental toxicity was observed [5]. Further, neurotoxicity and developmental toxicity was also reported for the fungicide tebuconazole. In animal studies, tebuconazole caused malformations in nervous system development, changes in brain morphometric parameters, and decreases in motor activity, for which no “No Observed Adverse Effects Level” dose was identified [54]. The EPA classifies tebuconazole as “Group C, possible human carcinogen” [54]. Peer reviewed studies reported that tebuconazole alters testosterone production and testicular morphometry [80, 81]. The European Union classifies tebuconazole as “suspected of damaging the unborn child”, and a 2014 review by the European Food Safety Agency noted that a classification of “may damage the unborn child” might be considered [82]. These effects are similar to the toxicity findings for the three pesticides described above and could warrant a 10X additional safety factor. In fact, when EPA scientists conducted the first FQPA assessment for this pesticide in 1998, they recommended a 10X FQPA safety factor for acute dietary exposures to tebuconazole for females age 13 and older and for infants and children [83]. In 2008, the EPA assigned a 3X FQPA factor for tebuconazole by referring to an unpublished benchmark dose analysis of toxicological studies for this pesticide [84], and that decision was maintained in a 2018 assessment [54].

Thiophanate-methyl, the fifth pesticide identified in this study that has an additional FQPA factor for chronic dietary exposures, is classified by the EPA as likely carcinogenic to humans, “based on thyroid tumors in rats and liver tumors in mice and evidence of aneugenicity” [56]. Endocrine disruption activity of thiophanate-methyl and its impacts on the thyroid and other endocrine pathways have been reported in peer-reviewed studies [85, 86]. In 2005, the EPA published a 3X FQPA safety factor for thiophanate-methyl for both acute and chronic dietary exposures, “due to an incomplete toxicity database” [87]; subsequently, the FQPA factor for acute dietary exposure to thiophanate-methyl was removed (reduced to 1X), while a 3X FQPA factor was retained for chronic dietary exposure, due to the lack of a developmental thyroid study [56]. The EPA has not required a developmental neurotoxicity study for thiophanate-methyl [56] and dismissed an argument that a 10X FQPA factor should be applied to this pesticide due to its endocrine disrupting effects [87]. It thus remains to be seen what the FQPA determination in the final EPA assessment for thiophanate-methyl will be.

### The FQPA factor determinations for organophosphates

Next, this study analyzed 12 organophosphate pesticides for which the FQPA determinations were made in 2015 and thereafter: acephate, bensulide, chlorethoxyfos, chlorpyrifos, diazinon, dicrotophos, dimethoate, ethoprop, malathion, phosmet, terbufos, and tribufos (Table 3). The choice of 2015 as the cut-off year is due to the 2015 publication of the EPA’s “Literature Review on Neurodevelopment Effects & FQPA Safety Factor Determination for the Organophosphate Pesticides” which stated that the FQPA 10X Safety Factor will be retained for organophosphates “for the population subgroups that include infants, children, youths, and women of childbearing age for all exposure scenarios” [88]. This decision was re-affirmed in an updated literature review on the topic, completed by the EPA in 2016 and released in 2017 [89].

Of the 12 organophosphates reviewed in this article, the 10X FQPA factor is applied in the latest published human health assessment documents for 11 chemicals, with the notable exception of chlorpyrifos (Table 3). For example, for acephate, the 10X uncertainty factor was applied “for infants, children, youth, and women of child-bearing age for all exposure scenarios due to uncertainty in the human dose-response relationship for neurodevelopmental effects” in a draft human health risk assessment published in 2018 [59]. The 10X FQPA factors were also assigned to the other ten organophosphates listed in Table 3 [60–69]. For chlorpyrifos, a human health risk assessment document published in 2016 applied a 10X FQPA factor [90], in line with the EPA’s latest policy documents for organophosphates. This decision was overturned in 2017 with the denial of a petition to revoke chlorpyrifos tolerances [58]. For this analysis, the FQPA determination deserves being quoted in its entirety:

“Dow AgroSciences submitted a comparative cholinesterase study (CCA) for chlorpyrifos. CCA studies are specially designed studies to compare the dose-response relationship in juvenile and adult rats. This CCA study includes two components: (1) Acute, single dosing in post-natal day 11 and young adult rats and (2) 11-days of repeating dosing in rat pups from [postnatal day] 11-21 and 11-days of repeated dosing in adult rats. The CCA study for chlorpyrifos is considered by EPA to be high quality and well-designed. The preliminary risk assessment for chlorpyrifos’ reports BMD estimates from this CCA study. Specifically, for the repeated dosing portion of the study, the BMD_10s_ of 0.80 (0.69 BMDL_10_) and 1.0 (0.95 BMDL_10_) mg/kg/day respectively for female pups and adults support the FQPA safety factor of 1X for the AChE inhibition endpoint used in the 2006 OP CRA [Cumulative Risk Assessment].” [58]

Thus, with a single paragraph, a 10X FQPA additional safety factor for chlorpyrifos was replaced with a 1X factor, a reversal in the overall trend of using a 10X FQPA factor for organophosphates. This decision is notable for several reasons. First, the text refers to a non-peer reviewed, non-published study from a company that manufactures chlorpyrifos. Second, this decision contradicts the EPA’s earlier position on the FQPA factor for organophosphates [88, 89]. Finally, it stands in stark contrast to peer-reviewed scientific literature and epidemiological studies demonstrating chlorpyrifos toxicity to infants and children [2, 91, 92]. As of January 2020, chlorpyrifos is classified as “not approved” in the European Union pesticides database [73], and it is scheduled for a phase out from the European market by April 2020 due to concerns about possible genotoxicity and developmental neurotoxicity associated with this pesticide.

### The FQPA assessments for non-dietary exposure pathways

In addition to dietary exposures, pesticide risk assessments consider other exposure pathways, such as incidental oral, inhalation, and dermal exposures that can contribute to aggregate exposure for a specific pesticide. These scenarios are relevant for pesticide exposures in the residential settings (both indoor and outdoor) such as pesticide applications on lawns and turf, insect repellent sprays, pet treatments, as well for incidental exposure from pesticide-treated materials, paints and preservatives [93]. Of note, restriction on the residential use of organophosphates is considered to be one of the accomplishments of the FQPA, such as the elimination of homeowner uses of chlorpyrifos in 2000 [94]. Non-dietary scenarios also apply to exposure from aerial drift of pesticides sprayed near places where children live, play, and study [95]. Risk assessment for exposure pathways other than diet may draw on toxicological studies specific to those exposure routes and the FQPA factor determinations may differ between different exposure scenarios.

For some pesticides analyzed in this study, the EPA assigned an FQPA factor for just one additional exposure pathway, or for no pathways other than dietary exposure. For example, for glyphosate there is an FQPA assessment for incidental oral exposure, with an FQPA factor of 1X. For other pesticides, the EPA published FQPA factors for multiple exposure pathways and duration scenarios. For example, for metolachlor, incidental oral exposure, dermal exposure and inhalation exposure scenarios all have an FQPA factor of 1X. Table 4 lists the non-organophosphate pesticides analyzed in this study for which the EPA assigned an FQPA factor for at least one non-dietary scenario, with a total of 30 pesticides and 80 non-dietary exposure scenarios. Pyrethroid insecticides are not included in this part of the analysis, as finalized human health risk assessments for the four pyrethroid insecticides analyzed in this study, bifenthrin, cypermethrin, fenpropathrin, and permethrin, are not yet available (see Discussion below). Overall, for incidental oral, dermal and inhalation exposures for non-organophosphate pesticides, additional FQPA safety factors were applied for 15, 31, and 41% of pesticides, respectively. The additional FQPA factor is most common for inhalation assessments; 11 out of 27 pesticides analyzed here have an FQPA factor greater than 1X for this exposure pathway (Table 4).

**Table 4:** The FQPA factor determinations for non-dietary exposures

	FQPA factor for incidental oral exposure	FQPA factor for dermal exposure	FQPA factor for inhalation exposure
Number of pesticides for which an FQPA factor was assigned for this exposure scenario	27	16	27
Number of pesticides for which this exposure scenario has an FQPA factor greater than 1X	4	5	11
Percentage of pesticides for which this exposure scenario has an FQPA factor greater than 1X	15%	31%	41%
Which pesticides have an FQPA factor greater than 1X for this scenario	Chlorpropham, glufosinate, tebuconazole, thiophanate-methyl	Chlorpropham, glufosinate, iprodione, tebuconazole, thiophanate-methyl	2,4-D, chlorothalonil, chlorpropham, cyprodinil, dicamba BAPMA salt, glufosinate, iprodione, tebuconazole, thiabendazole, thiophanate-methyl, trifloxystrobin
1,3-Dichloropropene	N/A	N/A	1X (4 scenarios for different exposure durations: acute, short-term; intermediate-term; and long-term)
2,4-D	1X (1 scenario for short- and intermediate-term)	N/A	10X (1 scenario for all exposure durations)
Acetamiprid	1X (2 scenarios for different exposure durations: short- and intermediate-term; and long-term)	1X (2 scenarios for different exposure durations: short- and intermediate-term; and long-term)	1X (1 scenario for short- and intermediate-term)
Azoxystrobin	1X (1 scenario for short- and intermediate-term)	N/A	N/A
Boscalid	1X (1 scenario for short- and intermediate-term)	1X (1 scenario for short- and intermediate-term)	1X (1 scenario for short- and intermediate-term)
Captan	1X (1 scenario for short-term)	1X (1 scenario for short- and intermediate-term)	1X (1 scenario for short- and intermediate-term)
Chlorothalonil	1X (2 scenarios for different exposure durations: short-term; and intermediate-term)	N/A	3X and 30X (2 scenarios for different exposure durations: acute inhalation with 3X FQPA factor; and short- and intermediate-term inhalation with 30X FQPA factor)
Chlorpropham	10X (1 scenario for short- and intermediate-term)	10X (1 scenario for short- and intermediate-term)	10X (1 scenario for short- and intermediate-term)
Clothianidin	1X (1 scenario for short-term)	1X (1 scenario for all durations)	1X (1 scenario for all durations)
Cyprodinil	1X (1 scenario for short-term)	N/A	10X (1 scenario for short- and intermediate-term)
Dicamba	1X (1 scenario for short- and intermediate-term)	N/A	2 scenarios: 1X for short-, intermediate- and long-term inhalation scenario for dicamba acid; 10X for short-, intermediate- and long-term inhalation scenario for dicamba BAPMA salt
Dimethomorph	1X (1 scenario for short-term)	N/A	N/A
Fenhexamid	1X (1 scenario for short- and intermediate-term)	N/A	1X (1 scenario for short- and intermediate-term)
Fludioxonil	1X (1 scenario for short- and intermediate-term)	N/A	1X (1 scenario for short- and intermediate-term)
Fluopicolide	1X (1 scenario for short- and intermediate-term)	1X (1 scenario for short- and intermediate-term)	1X (1 scenario for short- and intermediate-term)
Glufosinate	10X (1 scenario for short- and intermediate-term)	10X (1 scenario for short- and intermediate-term)	10X (1 scenario for acute, short- and intermediate-term)
Glyphosate	1X (1 scenario for short- and intermediate-term)	N/A	N/A
Imidacloprid	1X (1 scenario for all durations)	1X (1 scenario for all durations)	1X (1 scenario for all durations)
Iprodione	N/A	10X (1 scenario for dermal and inhalation, short- and intermediate-term)	
Metalaxyl/Mefenoxam	1X (1 scenario for short- and intermediate-term)	N/A	1X (1 scenario for short- and intermediate-term)
Metolachlor	1X (1 scenario for short-term)	1X (1 scenario for short- and intermediate-term exposure for children)	1X (1 scenario for short- and intermediate-term)
Paraquat	1X (1 scenario for short-term)	1X (1 scenario for short-term)	1X (1 scenario for short-term)
Pendimethalin	1X (1 scenario for short- and intermediate-term)	1X (1 scenario for short- and intermediate-term)	1X (1 scenario for short- and intermediate-term)
Pyraclostrobin	1X (1 scenario for short- and intermediate-term)	1X (1 scenario for short- and intermediate-term)	1X (1 scenario for short- and intermediate-term)
Pyrimethanil	N/A	1X (1 scenario for short- and intermediate-term)	1X (1 scenario for short- and intermediate-term)
Spinetoram	1X (1 scenario for short- and intermediate-term)	N/A	1X (1 scenario for short- and intermediate-term)
Tebuconazole	3X (1 scenario for short-term)	3X (1 scenario for short-term)	3X (1 scenario for short-term)
Thiabendazole	1X (2 scenarios, for short- and intermediate-term exposure for children and for short- and intermediate-term exposure for adults)	N/A	10X (1 scenario for short- and intermediate-term)
Thiophanate-methyl	3X (1 scenario for short- and intermediate-term)	3X (2 scenarios for short- and intermediate-term, and for long-term exposures)	3X (2 scenarios for short- and intermediate-term, and for long-term exposures)
Trifloxystrobin	1X (1 scenario for short-term)	N/A	10X (1 scenario for all durations)

*N/A* not available, indicating that an FQPA factor was not assigned for this exposure scenario

For five non-organophosphate pesticides with an additional FQPA safety factor for dietary exposures, an additional 10X factor was applied for other exposure scenarios for chlorpropham, glufosinate, and iprodione, and a 3X FQPA factor is applied for non-dietary exposure scenarios for tebuconazole and thiophanate-methyl. Among pesticides with a 1X FQPA factor for dietary exposures, 3X and 30X FQPA factors were applied, respectively, for the acute and repeated residential inhalation exposure scenarios for the fungicide chlorothalonil [21]. The FQPA factor of 30X was composed of 3X for the use of a LOAEL from the acute inhalation study (no NOAEL observed) and a 10X factor for the extrapolation of findings of an acute study to longer durations of exposure (see Additional file 1). The EPA describes chlorothalonil as “highly toxic via inhalation” and “Likely to be Carcinogenic to Humans” [21]; it is currently not approved for use in the European Union. The EPA also applied a 10X FQPA factor for inhalation exposures to the fungicides cyprodinil, thiabendazole, and trifloxystrobin, as well as for the herbicide 2,4-D and one form of the herbicide dicamba. These FQPA factors were assigned due to database uncertainties, such as a missing study or an extrapolation from a LOAEL to a NOAEL (see Additional file 1).

Finally, among the organophosphate pesticides listed in Table 3, 10X FQPA factors were assigned for non-dietary exposure scenarios for acephate, chlorethoxyfos, diazinon, dicrotophos, dimethoate, ethoprop, malathion, and tribufos [59–62, 65, 67–69]. For bensulide [63] and phosmet [64], 10X FQPA safety factors were assigned for all exposure pathways except for inhalation exposures, where a factor of 30X was applied due to “uncertainty in the human dose-response relationship for neurodevelopmental effects” and a lack of necessary inhalation studies for these pesticides.

## Discussion

The goal of this study is to stimulate discussion on the implementation of the children’s health safety factor in pesticide risk assessments. Overall, this analysis documented a rather limited use of additional FQPA factors in the EPA risk assessments of non-organophosphate pesticides. For acute dietary, chronic dietary, incidental oral, dermal and inhalation scenarios, respectively, 13, 12, 15, 31 and 41% of reviewed pesticides have an additional FQPA factor for these exposure pathways. These statistics are similar to what was reported in the first decade after the FQPA passage [5, 8–11]. Importantly, even if an additional FQPA factor is assigned, it does not necessarily represent children’s health protection; for non-organophosphate pesticides, the EPA’s primary rationale for applying an additional FQPA factor is due to data uncertainties.

In 2006, the EPA Office of Inspector General wrote that significant challenges remain in the FQPA implementation and that addressing these challenges could improve children’s health [96]. For example, the Office of Inspector General noted that the EPA’s required testing for pesticide registrations “does not include sufficient evaluation of behavior, learning, or memory in developing animals.” In view of the author, the EPA has not yet succeeded in addressing fine neurological changes that may occur following pesticide exposures, as exemplified in the case of chlorpyrifos where high quality data from human studies were dismissed in favor of a mechanistic study conducted by the pesticide manufacturer [58].

Over the course of this research project, new EPA documents relevant to the FQPA determinations have been published. First, the EPA’s “Re-Evaluation of the FQPA Safety Factor for Pyrethroids” released in July 2019 stated that the FQPA safety factor for pyrethroids can be reduced to 1X for all populations [29]. This approach would remove the 3X FQPA safety factors that were previously applied for pyrethroid exposures for children 6 and younger for scenarios such as acute dietary exposures, incidental oral exposures, dermal, and inhalation exposures [97–99]. As of January 2020, the EPA’s proposal on the FQPA determination for pyrethroids has not yet been finalized and remained open for public comment. However, the re-evaluation document gives an indication of the EPA’s projected removal of FQPA factors for this group of insecticides. Since final determinations are not yet available, pyrethroids were not included in the analysis of non-dietary exposure pathways presented in this study; if the EPA finalizes the proposal of applying a 1X FQPA factor to pyrethroids, this would further decrease the frequency of the FQPA factor application for non-dietary exposure scenarios.

Second, two recent EPA assessments for the herbicide metolachlor are now available for comparison, from 2018 [100] and 2019 [15]. In both documents, a 1X FQPA factor was applied for all exposure scenarios. Yet, in the 2019 assessment, the EPA proposed to establish the reference dose for metolachlor based on an older rather than a newer animal toxicology study, which would increase the exposure limit to metolachlor by 2.7-fold [15]. In risk assessments published in 1995, 2014 and 2018, the chronic reference dose for metolachlor was based on a 1-year toxicity study in dogs conducted in 1989, with the reported No Observed Adverse Effect Level (NOAEL) of 9.7 mg/kg/day [100]. The same study and the same NOAEL have been used in the European Union assessment of metolachlor and the development of the European “acceptable daily intake” dose for chronic exposures to this chemical [101]. Rather than requiring a new, more refined toxicology study, in the 2019 assessment for metolachlor the EPA used a 1981 study on rats, with a reported NOAEL of 26 mg/kg/day, resulting in a higher chronic reference dose [15]. This decision diverges from the EPA’s own guidance on the FQPA implementation which emphasized the identification of the most sensitive toxicity effects for reference dose development [3].

This study raises two themes for future research and public policy: first, the inclusion of human data whenever available; and second, the development of novel approaches for assessing pre- and postnatal toxicity. The importance of both topics was noted in earlier reviews [5, 102]. In a landmark 1993 report, “Pesticides in the Diets of Infants and Children” [103], the National Research Council highlighted the vulnerability of infants and children to pesticides, setting the groundwork for the Food Quality Protection Act. Five years after the passage of the FQPA, the Centers for Disease Control and Prevention (CDC) published their first report documenting the presence of organophosphate pesticide metabolites in urine [104]. Since that time, biomonitoring studies conducted by the CDC and by independent researchers reported the presence of multiple pesticides and their metabolites in the American population, including the herbicides 2,4-D and glyphosate; the neonicotinoid insecticides acetamiprid, clothianidin and imidacloprid; organophosphate and pyrethroid insecticides; and fungicide metabolites [105–108]. In 2006, the National Research Council wrote that biomonitoring has become central to “identifying, controlling, and preventing population exposures to potentially harmful environmental chemicals” [109]. More research is needed on pesticide exposures for children under 6, since the CDC biomonitoring program focuses on populations 6 years of age and older [105, 110], and on the methods for including biomonitoring data in pesticide risk assessments.

In 2016, the EPA completed a “Framework for Incorporating Human Epidemiologic & Incident Data in Risk Assessments for Pesticides” which noted that toxicology and risk assessment are undergoing transformational changes driven by new research [111]. One such development relevant to risk assessment is the identification of Key Characteristics applicable to endocrine disruption [112], male and female reproductive toxicity [113, 114] and carcinogenesis [115, 116]. The Key Characteristics approach highlighted the diversity of toxicity pathways that can lead from exposure to environmental contaminants to an elevated risk of disease. It remains to be seen how the EPA would incorporate these new approaches into regulatory risk assessment for cancer [117, 118], identification of endocrine disruptors [119], and risks to children’s health.

## Conclusions

The use of an additional 10-fold margin of safety for children’s health protection remains highly relevant for pesticide risk assessments in light of the ever-growing research on the human health impacts of environmental contaminants and new exposure data generated by biomonitoring studies. Such a health-protective approach is especially important for pesticides that can cause harm to the nervous system, hormonal disruption and cancer. In the meanwhile, the limited application of the FQPA factor in pesticide risk assessments is a missed opportunity for the EPA to fully use the authority of the Food Quality Protection Act for protecting children’s health.

## Supplementary information

**Additional file 1:** Rationale for the additional FQPA factors for non-organophosphate pesticides. Citations from EPA documents posted on the Regulations.gov.

## Data Availability

This study used publicly accessible information from the U.S. Government website Regulations.gov.

## Abbreviations

2,4-D: 2,4-dichlorophenoxyacetic acid
BMD: Benchmark dose
BMDL: Benchmark dose lower confidence limit
CDC: Centers for disease control and prevention
EPA: U.S. Environmental Protection Agency
FQPA: Food quality protection act of 1996
LOAEL: Lowest observed adverse effect level
NOAEL: No observed adverse effect level
UF: Uncertainty factor
USDA: U.S. Department of Agriculture
USGS: U.S. Geological Survey
