# Supplementary material for: Application of the Food Quality Protection Act children’s health safety factor in the U.S. EPA pesticide risk assessments
Source: Environ Health. 2020 Feb 10;19:16. doi: 10.1186/s12940-020-0571-6 (PMC7011289; doi:10.1186/s12940-020-0571-6)
Supplement: Supplementary file 1 — Additional file 1: Rationale for the additional FQPA factors for non-organophosphate pesticides. Citations from EPA documents posted on the Regulations.gov. [file 12940_2020_571_MOESM1_ESM.docx]

**Additional File for “Application of the** **Food Quality Protection Act Children’s Health Safety Factor in the U.S. EPA Pesticide Risk Assessments.”**

**Rationale for the additional FQPA factors for non-organophosphate pesticides.**

| **Pesticides, in alphabetical order** | **EPA citation and reference** | **Why the FQPA factor is applied** |
| --- | --- | --- |
| 2,4-D | “For the inhalation exposure assessments for short- and intermediate-term durations, a total uncertainty factor of 300X is appropriate [3X for interspecies extrapolation (reduced from 10X because RfC methodology was used, which takes into consideration the pharmacokinetic differences between animals and humans), 10X for intraspecies variation, and a 10X FQPA database uncertainty factor (for extrapolation from a LOAEL to a NOAEL)].” [19] | Uncertainty factor (UF_LOAEL→NOAEL_) due to use of LOAEL to extrapolate a NOAEL |
| Azoxystrobin | “The Food Quality Protection Act (FQPA) Safety Factor (SF) has been reduced to 1X for non-acute exposure durations and non-inhalation exposure routes. For assessing acute risk, EPA is retaining a FQPA safety factor (SF) of 3X to account for the use of a lowest observed adverse effect level (LOAEL) from the acute neurotoxicity study to derive an acute reference dose.” [30] | Due to the use of LOAEL for setting the reference dose |
| Chlorothalonil | “For inhalation exposure scenarios, an acute inhalation study was used with a composite FQPA SF of 30X applied to short- and intermediate-term residential inhalation exposures on the basis that there was no inhalation study of appropriate duration (only acute studies are available) available for assessing risk from  repeated inhalation exposures. The composite FQPA SF of 30x was made up of 3X for the use of a minimal LOAEL (no NOAEL achieved) from the acute inhalation study and a 10X factor for the extrapolation of findings of an acute study to longer durations of exposure.” [22] | Due to the use of LOAEL from an acute inhalation study for assessing inhalation exposures |
| Chlorpropham | “For chlorpropham, the 10X FQPA SF has been retained for all exposure scenarios to account for the UFDB that was applied due to concerns that chlorpropham has the potential to disrupt thyroid function during potentially sensitive lifestages (pregnancy, prenatal, and postnatal periods). There is no evidence of increased susceptibility to offspring in the developmental or reproduction toxicity studies; however, chlorpropham was found to cause thyroid toxicity in dogs and there is a lack of data on whether pregnant animals or fetuses are more or less susceptible than adult animals to the impact of chemicals that alter thyroid hormone homeostasis.” [35] | Database uncertainty factor (UFDB) and concern about thyroid toxicity |
| Cyprodinil | “For inhalation exposure scenarios for all population groups, EPA is retaining a 10X FQPA safety factor for the lack of a route specific inhalation study.” [37] | Data gap due to a missing study |
| Dicamba BAPMA (N,N-Bis-(3-aminopropyl) methylamine) | “The 10X FQPA SF is retained for assessing inhalation risks for the dicamba BAPMA salt, the FQPA SF is retained in the form of a LOAEL to NOAEL factor (UF_L_) since the [Point of Departure] used was a LOAEL.” [24] | Due to the use of LOAEL to extrapolate a NOAEL |
| Dimethomorph | “…recommended that the 10X FQPA Safety Factor (SF) be retained for acute dietary exposure scenario for extrapolation of a NOAEL from a LOAEL. For other exposure scenarios, the FQPA SF is reduced to 1X since there is no evidence of increased qualitative or quantitative susceptibility in the young and exposure estimates are unlikely to underestimate risk. There is a 10X database uncertainty factor applied to occupational inhalation exposure scenario due to a database deficiency of a subchronic inhalation study. However, this database uncertainty factor is not considered a FQPA factor since it is only applied to occupational inhalation exposure scenario..” [39] | Uncertainty factor (UF_L_) due to the use of LOAEL to extrapolate a NOAEL |
| Glufosinate | “An additional uncertainty factor of 10X was used for other exposure scenarios (total uncertainty factor of 1000X) because the studies selected for endpoint setting used LOAELs (the developmental neurotoxicity and the 28-day inhalation studies) and did not establish NOAELs.” “When the DNT [Developmental Neurotoxicity] study is used as an endpoint for risk assessment, an extra 10X database uncertainty (UF_L_) factor is applied because the DNT study did not demonstrate a NOAEL for altered brain morphometrics. This additional uncertainty factor will also account for indications of increased qualitative or quantitative sensitivity evident in the rat and rabbit developmental studies, and the rat multigenerational reproduction study.” [23] | Uncertainty factor (UF_L_) due to the use of LOAEL to extrapolate a NOAEL |
| Iprodione | “The most sensitive endpoint in the iprodione database is the reduction of testosterone. The point of departure (POD) used for all exposure risk assessments was selected from a male rat pubertal assay, based on reduced serum levels of testosterone. An uncertainty factor of 1000X was applied to the endpoint selected for all routes and durations of exposure (10X for interspecies extrapolation, 10X for intraspecies variation, 10X FQPA SF). The FQPA safety factor accounts for the lack of a NOAEL in the testosterone data (UF_LOAEL→NOAEL_) and for the lack of the guideline studies (reproduction study, 870.3800 and 28-day inhalation toxicity study, 870.3465).” [49] | Uncertainty factor (UF_LOAEL→NOAEL_) due to use of LOAEL to extrapolate a NOAEL |
| Tebuconazole | “The data from prenatal developmental toxicity studies in mice and a developmental neurotoxicity study in rats indicated an increased quantitative and qualitative susceptibility following in utero exposure to tebuconazole.” “Tebuconazole demonstrated neurotoxicity in the acute neurotoxicity study in rats; the lowest observable adverse effect level (LOAEL) of 100 mg/kg/day was based on increased motor activity in male and female rats and decreased footsplay in female rats. Although the subchronic neurotoxicity study was unacceptable since there was inadequate dosing, a new subchronic neurotoxicity study is not needed to evaluate levels at which subchronic neurotoxicity might occur; neurotoxicity was seen in other studies in the database at considerably lower doses than those tested in the subchronic neurotoxicity study. Malformations indicative of nervous system development disruption were seen in developmental toxicity studies in mice, rats, and rabbits. Neurotoxicity was also seen in the rat developmental neurotoxicity study as decreases in body weights, decreases in absolute brain weights, changes in brain morphometric parameters, and decreases in motor activity in offspring at the LOAEL of 8.8 mg/kg/day; a no observable adverse effect level (NOAEL) could not be established. The LOAEL (8.8 mg/kg/day) was employed as the point of departure (POD) for assessing risk for all exposure scenarios, and an FQPA SF of 3X has been retained as an uncertainty factor for use of a LOAEL to extrapolate a NOAEL (UF_L_). [56] | Uncertainty factor (UF_L_) due to the use of LOAEL to extrapolate a NOAEL |
| Thiabendazole | “…recommends reducing the FQPA Safety Factor (SF) to 1X for all scenarios with the exception of the inhalation scenario where the FQPA SF is retained  at 10X in the form of a database uncertainty factor (UF_DB_) for lack of a subchronic inhalation study with thyroid measurements.” [57] | Database uncertainty factor (UFDB) due to a missing study |
| Thiophanate-methyl | “For chronic dietary and non-dietary (residential) exposure, the Food Quality Protection Act (FQPA) Safety Factor was reduced to a 3X due to a data gap for a developmental thyroid study.” [58] | Data gap due to a missing study |
| Trifloxystrobin | “A 10X database UF/FQPA has been retained for inhalation endpoints only to account for the lack of the subchronic inhalation toxicity study for trifloxystrobin at this time.” [59] | Database uncertainty factor (UFDB) due to a missing study |
